# Can Epirubicin Be Used in Laryngology Practice Like Mitomycin? An Experimental and Pioneering Study

**DOI:** 10.22038/ijorl.2025.87024.3938

**Published:** 2025

**Authors:** Erdem Koroglu, Selahattin Genc, Serdar Baser, Ferit Bayakır, Ahmet Tugrul Eruyar, Busra Yaprak Bayrak

**Affiliations:** 1 *University of Health Sciences Kocaeli Derince Health Research and Application Center, Otorhinolaryngology and Head & Neck Surgery Clinic. *; 2 *Kocaeli University Faculty of Medicine, Pathology Clinic. *

**Keywords:** Epirubicin, Mitomycin-C, Laryngeal Posterior Commissure, Laryngostenosis

## Abstract

**Introduction:**

Epirubicin and mitomycin-C share similar mechanisms of action, with both exhibiting antiproliferative effects by inhibiting DNA and protein synthesis. While the efficacy of mitomycin-C in laryngology is well established, this study aims to investigate whether epirubicin can produce comparable clinical outcomes in this field.

**Materials and Methods:**

Ten rabbits were included in this experimental study. A thermal injury was created at the posterior commissure using a conchal probe. Following the injury, one group was treated with mitomycin-C, while the other received epirubicin. After a six-week post-treatment period, the rabbits were euthanized, and both macroscopic and microscopic evaluations were performed to assess stenosis, scarring, granulation tissue, necrosis, and ulceration. Two pathologists, blinded to the treatment groups, independently examined the histological samples.

**Results:**

Macroscopically, no significant differences were observed between the two groups in terms of scarring, synechiae, or granulation tissue formation at the posterior commissure. However, the mitomycin-C group demonstrated a relatively milder tissue response. Microscopic analysis revealed grade 3 collagen deposition in one rabbit and grade 1 in two rabbits from the epirubicin group. In comparison, the mitomycin-C group showed grade 1 deposition in two rabbits and grade 2 in another two. The average fibroblast count was 83.3 in the epirubicin group versus 59 in the mitomycin-C group.

**Conclusions:**

Although this pioneering study does not provide conclusive evidence that epirubicin is as effective as or superior to mitomycin-C in laryngology, it highlights epirubicin’s potential as a promising candidate for further investigation in the treatment of laryngeal conditions.

## Introduction

Laryngology, a subspecialty within otolaryngology, often requires innovative therapeutic strategies to manage conditions such as laryngeal stenosis and scarring. Laryngostenosis refers to the narrowing of the airway within the larynx, resulting from various etiologies, including post-surgical fibrosis, trauma, or chronic inflammation (1,2). 

Current treatment modalities, such as surgical intervention and the use of mitomycin-C, offer variable success rates and often necessitate repeated procedures with prolonged recovery periods. The demand for novel therapeutic agents that can effectively regulate scar formation and enhance mucosal healing is increasingly emphasized in laryngology.

Mitomycin-C has been established as an effective agent in reducing fibrosis and promoting mucosal repair following laryngeal surgery (3,4). It is currently used in a wide range of procedures involving stenosis correction in the lacrimal sac, larynx, trachea, choanal atresia, and esophageal strictures (5–8).

Nonetheless, the search for alternative agents that offer comparable or superior outcomes continues. Epirubicin is a well-established chemotherapeutic agent, particularly in the treatment of breast cancer, due to its potent ability to inhibit cell proliferation and induce apoptosis in rapidly dividing cells (9).

Its antiproliferative effects arise from its interference with DNA and RNA synthesis, similar to those of mitomycin-C. Given this shared mechanism of action, it is hypothesized that epirubicin may also prove effective in managing laryngeal conditions characterized by excessive fibroproliferation and scarring. This study evaluates the potential of epirubicin in laryngology and compares its effects with those of mitomycin-C.

## Materials and Methods

This study was conducted by researchers certified in animal experimentation, with approval obtained from the local ethics committee (koü hadyek 4/3-2017). The authors affirm that all procedures adhered to national and institutional ethical guidelines concerning the care and use of laboratory animals.

*Generation of posterior glottic damage and its treatment with the active ingredient*

**Fig 1:**
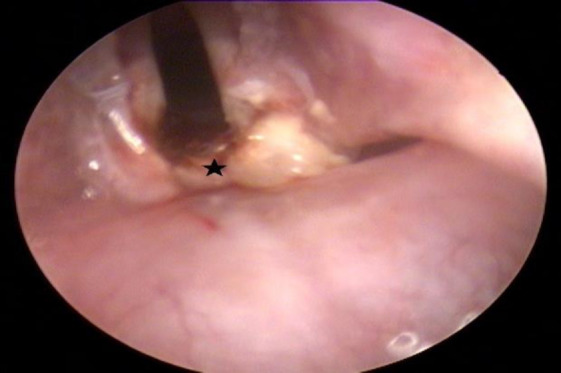
Thermal injury was inflicted on the posterior commissure, including the medial facets of the arytenoid cartilages and the posterior 1/3 of the membraneous vocal cord. Direct laryngoscopic view. Asteriks; posterior commissure.

Ten 12-week-old white New Zealand rabbits, weighing between 2.1 and 2.4 kg, were included in the study. General anesthesia was achieved via intramuscular administration of xylazine (5 mg/kg) and ketamine (35 mg/kg). A metal tongue depressor was bent into an L-shape to retract the tongue, enabling visualization of the posterior glottis with a 0°, 4 mm rigid endoscope (Karl Storz GmbH & Co., Tuttlingen, Germany). A conchal probe from a coblator device (Coblator II, ArthroCare Corporation, USA) was used to induce thermal injury involving the posterior commissure, medial facets of the bilateral arytenoid cartilages, and the posterior third of the membranous vocal folds (Figure 1).

Following the injury, the animals were randomly divided into two groups (n=5 per group). The first group received mitomycin-C (Mit-C), and the second group was treated with epirubicin (Ep). Mit-C powder was dissolved in injection-grade sterile water to a concentration of 0.4 mg/ml, based on literature-defined safe and effective doses. Epirubicin was diluted from a 50 mg/25 ml vial to a concentration of 0.5 mg/ml using sterile water, as no prior laryngological studies had established an appropriate concentration; this dose was therefore empirically estimated. The respective drug solutions were applied to cotton pads and placed on the injured posterior glottis for five minutes. To mitigate potential adverse effects such as necrosis, the treated area was subsequently rinsed with water-soaked cotton pads. All animals were housed in a certified animal laboratory under the supervision of a veterinary surgeon until the end of the sixth week, when they were euthanized. Euthanasia was performed using induction anesthesia followed by intracardiac injection of a lethal dose of pentobarbital. Just prior to euthanasia, a 0°, 4 mm rigid endoscope was used to perform direct laryngoscopy and assess the posterior commissure for stenosis, scarring, granulation tissue, necrosis, and ulceration, as evaluated by an otolaryngologist.

*Macroscopic and microscopic evaluation*

Following euthanasia, a midline cervical incision was made using a #22 scalpel blade to expose the larynx. 

The laryngeal framework, including the hyoid bone, proximal tracheal rings, and adjacent esophagus, was excised en bloc. The specimens were fixed in 10% formalin for 24 hours. Following standard alcohol dehydration and paraffin embedding, 5-micrometer-thick tissue sections were prepared for analysis. Two pathologists, blinded to group allocation, independently performed histopathological assessments. Hematoxylin-eosin-stained slides were evaluated for fibroblast proliferation, capillary angiogenesis, leukocyte infiltration, submucosal gland loss, and re-epithelialization. Masson Trichrome staining was used to assess collagen deposition, which was graded on a scale from 1 to 3 at 40x magnification. Fibroblast counts were performed at 400x magnification in areas of highest density. Leukocyte infiltration was graded on a 0–3 scale (Grade 0: none; Grade 1: mild; Grade 2: moderate; Grade 3: severe) at 40x magnification. Capillary angiogenesis was assessed at 200x magnification in the most vascularized regions.

## Results

A total of ten rabbits were enrolled at the beginning of the study, with five allocated to the mitomycin-C (Mit-C) group and five to the epirubicin (Ep) group. One rabbit in the Mit-C group (1/5, 20%) died on the second day after local drug administration. In the Ep group, two rabbits (2/5, 40%) died during the first- and third-weeks post-application. The deceased rabbit in the Mit-C group exhibited markedly edematous vocal cords, with airway obstruction identified as the probable cause of death. In contrast, the two deceased animals in the Ep group had unobstructed airways, and the causes of death remained undetermined. As a result, the final analysis was conducted on four rabbits in the Mit-C group and three in the Ep group.

*Direct laryngoscopy and Macroscopic findings*

Scarring, synechiae, ulceration, and granulation tissue formation were assessed via serial imaging and telescopic inspection. No statistically significant differences were observed between the Ep and Mit-C groups in terms of scarring, synechiae, ulceration, or granulation tissue formation affecting the medial facets of the arytenoid cartilages, interarytenoid areas, or posterior membranous vocal folds. However, the Mit-C group exhibited a relatively milder tissue response overall (Figures 2a and 2b).

**Fig 2a:**
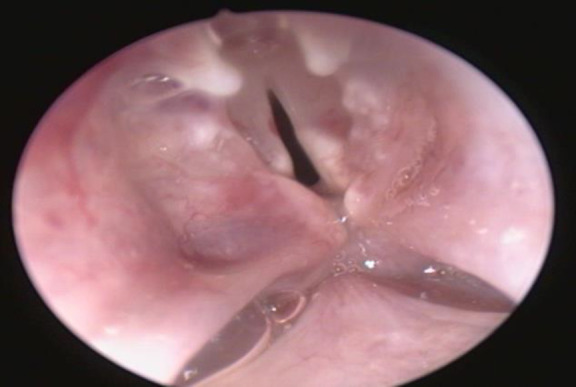
A healthy larynx sample where mitomycin- C was applied. Direct laryngoscopic view, just before scarification

**Fig 2b:**
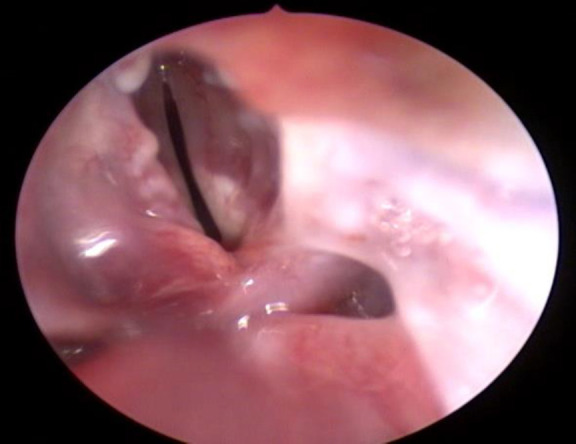
A larynx sample where epirubicin was applied. Despite the subtle hyperemia of the posterior commissure, the larynx seemed similar to normal. Direct laryngoscopic view, just before sacrification

*Microscopic findings*

In the Ep group, collagen deposition was graded as 3 in one rabbit (n = 1/3; 33.3%) and grade 1 in the remaining two (n = 2/3; 66.7%) (Figure 3a). In the Mit-C group, two animals (n =2/4; 50%) showed grade 1 collagen deposition, while the other two exhibited grade 2 (n = 2/4; 50%).

**Fig 3a:**
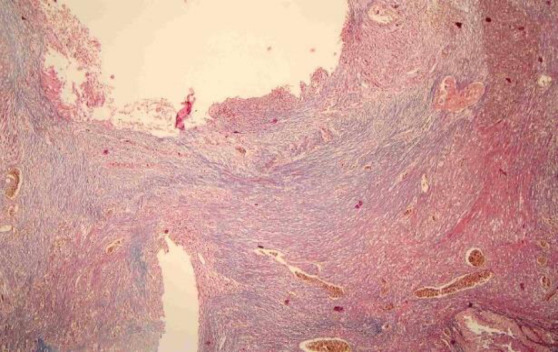
Grade 3 collagen deposition where collagen is depicted blue (Masson-Trichrome, x40). The slide was prepared from the specimen that received epirubicin and displayed exuberant reaction

**Fig 3b:**
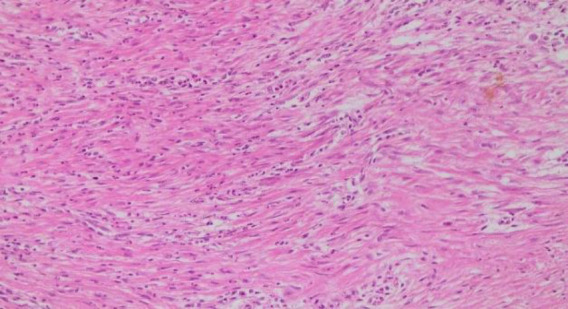
The same sample, H-E stain. Intense fibroblastic activity is evident (x200)

Submucosal gland loss was observed in one animal (n = 1/3; 33.3%) in the Ep group, while the remaining two did not exhibit this finding (n = 2/3; 66.7%). Similarly, one rabbit in the Mit-C group had submucosal gland loss (n = 1/4; 25%), and three did not (n = 3/4; 75%).

No evidence of re-epithelialization was seen in any rabbit in the Ep group (n = 0/3; 0%), whereas one rabbit in the Mit-C group (n = 1/4; 25%) exhibited re-epithelialization.

Capillary angiogenesis was noted in two rabbits in the Ep group (n = 2/3; 66.7%) and one rabbit in the Mit-C group (n = 1/4; 25%).

In terms of leukocyte infiltration, one rabbit in the Ep group (n = 1/3; 33.3%) demonstrated grade 3 infiltration, while the other two showed no infiltration (n = 2/3; 66.7%). 

In the Mit-C group, three rabbits (n = 3/4; 75%) had grade 1 infiltration, and one had grade 2 (n = 1/4; 25%). Fibroblast counts were markedly elevated in one rabbit from the Ep group (Figure 3b). The other two rabbits in the Ep group had fibroblast counts comparable to or lower than those in the Mit-C group. A summary of the histological findings is presented in Table 1.

**Table 1:** Groups and histopathologic findings

**Subjects**	**Collagen deposits (Grade)**	**Loss of submucosal glands**	**Complete re-epithelialization**	**Capillary angiogenesis x200**	**Leukocyte infiltration (Grade)**	**Fibroblast count x400**
Ep 1	3	+	-	52	3	175
Ep 2	1	-	-	21	0	56
Ep 3	1	-	-	15	0	19
Mit- C 1	2	+	+	14	1	76
Mit- C 2	2	-	-	19	1	82
Mit- C 3	1	-	-	12	1	45
Mit- C 4	1	-	-	14	2	33

Ep: Epirubicin group; Mit– C: Mitomycin– C group.

## Discussion

The management of laryngeal stenosis continues to present a significant clinical challenge, necessitating the investigation of novel therapeutic agents capable of reducing scar formation and promoting mucosal healing. This study sought to compare the effects of epirubicin and mitomycin-C on laryngeal tissue following induced injury in a rabbit model. 

The findings suggest that, although both agents possess therapeutic potential, their effects differ in terms of histological and tissue response. Mitomycin-C has long been recognized for its efficacy in reducing fibrosis and promoting healing in various surgical contexts, including laryngeal interventions (10). Consistent with prior literature, our results showed that the Mit-C group exhibited a milder tissue response, with lower collagen deposition and fibroblast proliferation than the epirubicin group. This reinforces the established role of mitomycin-C in suppressing excessive fibroblast activity and limiting scar tissue formation. However, the airway obstruction and subsequent mortality in one Mit-C-treated rabbit underscore the need for careful application and monitoring, particularly in anatomically delicate areas such as the larynx. Epirubicin shares a similar mechanism of action with mitomycin-C, prompting the question of whether it could exert comparable clinical effects within the larynx. This study represents the first attempt in the literature to address that question. Given the lack of prior data and ethical considerations regarding animal use, we intentionally limited the number of animals involved. Nevertheless, epirubicin demonstrated an intriguing profile in modulating the healing process, particularly through its effects on collagen deposition and fibroblast activity, which align with its known antiproliferative effects in oncology.

However, the observation of submucosal gland loss in some epirubicin-treated animals raises concerns regarding its long-term impact on laryngeal function and tissue integrity. While these preliminary results suggest that epirubicin may offer benefits in managing laryngeal scarring, additional studies are required to establish its safety profile and therapeutic utility.

The histological findings further indicate that epirubicin may provoke a more active fibroblast response. A heightened fibroblast count is typically associated with robust wound healing, suggesting that epirubicin might not only limit excessive fibrosis but also promote more organized tissue regeneration. Clarifying the nature of fibroblast modulation by epirubicin may help refine its use in clinical practice.

Interestingly, no significant differences in macroscopic outcomes were noted between the treatment groups. This raises important questions regarding the timing and dosing of drug application. It is possible that the six-week observation period was insufficient to capture long-term or late-phase tissue changes. Future studies should therefore consider extended follow-up to assess functional recovery, including voice quality. Moreover, evaluating different epirubicin concentrations and routes of administration could aid in defining its optimal therapeutic window and allow for more direct comparison with mitomycin-C protocols.

Regarding inflammatory markers, no meaningful differences in leukocyte infiltration or angiogenesis were observed between the groups, implying that both agents may exert comparable anti-inflammatory effects in this setting. Nonetheless, the divergent collagen and fibroblast profiles suggest that the underlying mechanisms of tissue remodeling may differ between epirubicin and mitomycin-C. Epirubicin’s apparent ability to both regulate inflammation and promote fibroblast activity further supports its dual potential in wound modulation.

Submucosal gland loss was observed in one rabbit from each group, and re-epithelialization was only noted in one rabbit in the Mit-C group. While these findings alone are insufficient to draw definitive conclusions, they suggest that epirubicin may have similar efficacy to mitomycin-C in certain histological parameters.

As for angiogenesis and leukocyte infiltration, milder inflammatory cell infiltration and more evident capillary formation were observed in the Ep group. However, previous studies have shown that these two parameters are not reliable indicators of clinical efficacy for mitomycin-C (11). Therefore, their contribution to differentiating between the two agents in this context remains limited. The rabbit model used in this study provides a valuable framework for examining post-injury healing in the larynx. However, extrapolating these results to human clinical scenarios should be done cautiously. Future research should include long-term outcomes and possibly explore combination therapies that could enhance epirubicin’s effects while minimizing adverse events.On macroscopic inspection, no posterior glottic ulcers, necrosis, webs, or granulation tissue were noted in the Mit-C group. The Mit-C-treated larynges appeared more stable and morphologically preserved. In contrast, one rabbit in the Ep group exhibited significant scar formation. The suboptimal macroscopic results in the Ep group may be attributed to an excessively high drug concentration. Unlike mitomycin-C, for which well-documented safe dosing ranges exist, epirubicin has not yet been optimized for laryngological use. A separate pilot study to determine optimal epirubicin dosing might have yielded more refined results; however, such an approach would have necessitated the use of additional animals. To ethically limit animal use, we applied a half-diluted concentration to an initial group of five rabbits. It is possible that even this reduced concentration was too high, resulting in tissue necrosis and subsequent fibrosis.

This study has certain limitations. Notably, the absence of experimental subgroups aimed at optimizing epirubicin dosing is a methodological shortcoming. Additionally, the poor histological and macroscopic outcomes observed in one particular rabbit in the Ep group disproportionately influenced overall group comparisons. 

While exclusion of this outlier might have improved data clarity, it would have further reduced the already small sample size. Future studies should consider larger sample sizes and the inclusion of a control group that does not receive any active agent. Such a design would facilitate clearer interpretation of each agent's effects relative to untreated healing.

Finally, future research should incorporate patient-centered outcome measures. Evaluating parameters such as voice quality and treatment satisfaction would provide a more comprehensive understanding of the clinical relevance of epirubicin use in laryngology. Incorporating patient perspectives will strengthen the translational value of subsequent findings and ensure that therapeutic developments align with real-world patient needs. As the field continues to evolve, epirubicin may emerge as a valuable addition to the laryngologist’s therapeutic arsenal, potentially enabling more tailored and effective patient care.

## Conclusions

In conclusion, this experimental study adds to the growing body of literature exploring epirubicin as a potential alternative to mitomycin-C in the treatment of laryngeal conditions characterized by fibrosis and scarring. 

While both agents demonstrated promising histological effects, their mechanisms of action and safety profiles may differ. Further research is needed to identify optimal dosing strategies, evaluate long-term safety, and clarify clinical efficacy. Careful consideration of the risks and benefits of each agent will be essential in the development of safe, effective, and patient-centered treatments for laryngeal pathologies.
